# Usefulness of Immature Granulocytes as A Prognostic Factor in ST-Elevation Myocardial Infarction

**DOI:** 10.21470/1678-9741-2021-0088

**Published:** 2022

**Authors:** Mustafa Korkut, Cihan Bedel, Ramazan Sivil, Mücahit Alp Arslan, Fatih Selvi, Görkem Kuş, Ökkeş Zortuk

**Affiliations:** 1 Department of Emergency Medicine, Health Science University Antalya Training and Research Hospital, Antalya, Turkey.; 2 Department of Public Health, Iğdır Public Health Center, Iğdır, Turkey.; 3 Department of Cardiology, Health Science University Antalya Training and Research Hospital, Antalya, Turkey.

**Keywords:** Granulocytes, Inflammation, ST-Segment Elevation Myocardial Infarction, Disease Management

## Abstract

**Objective:**

ST-segment elevation myocardial infarction (STEMI) is a serious, life-threatening disease. Inflammatory markers have recently become the focus of attention in the assessment of severity in the early stages of STEMI. This study aimed to evaluate the importance of immature granulocytes (IG) as a prognostic marker in STEMI.

**Methods:**

Patients admitted to the coronary care unit with a diagnosis of STEMI and who underwent primary percutaneous coronary intervention (pPCI) within the period from January 1, 2019 to January 1, 2020, were retrospectively scanned. A total of 146 patients were analised; of these, 112 (76.7%) were male and 34 (33.3) were female, with a mean age of 62.65±14.06 years. Patients’ age, gender, haemogram, biochemistry, and mortality results were recorded. The patients were divided into two groups as low (<0.6) and high (≥0.6) IG levels and compared.

**Results:**

The mean IG levels were significantly higher in the non-survivor group compared to the survivor group (1.12±0.22 vs. 0.50±0.28, P<0.001). Mortality rates were significantly higher in the high IG group compared to the low IG group (26.9% vs. 9.6%, P=0.006). IG was shown to predict mortality with a sensitivity of 72.2% and a specificity of 77.8% at a cut-off value of 0.65 (area under the curve: 0.740, 95% CI: 0.635-0.846, P<0.001).

**Conclusion:**

High IG values in the blood collected at the time of admission to the emergency department are a marker of mortality in patients with STEMI.

**Table:** Abbreviations, Acronyms & Symbols

ALT	= Alanine transaminase	LVEF	= Left ventricular ejection fraction
BUN	= Blood urea nitrogen	MI	= Myocardial infarction
CBC	= Complete blood count	MPV	= Mean platelet volume
CRP	= C-reactive protein	NLR	= Neutrophil-lymphocyte ratio
DBP	= Diastolic blood pressure	NT-proBNP	= N-terminal pro-brain natriuretic peptide
EF	= Ejection fraction	OR	= Odds ratio
Hb	= Hemoglobin	Plt	= Platelets
HDL	= High-density lipoprotein	pPCI	= Primary percutaneous coronary intervention
hsTnT	= High-sensitive troponin-T	ROC	= Receiver operating characteristic
HT	= Hypertension	SBP	= Systolic blood pressure
IG	= Immature granulocytes	STEMI	= ST-segment elevation myocardial infarction
LDL	= Low-density lipoprotein	WBC	= White blood cell

## INTRODUCTION

ST-segment elevation myocardial infarction (STEMI) is caused by a blockage in the coronary arteries and, as a result, interruption of blood flow to the myocardium^[1,2]^. Despite the significant improvements achieved so far through modern technological advances and revascularization techniques, medical treatments and secondary prevention measures, STEMI remains a major cause of mortality not only in our country, Turkey, but also in the world^[3]^. In-hospital mortality and potential prognostic indicators after a myocardial disease have been investigated in many studies^[4]^. Advancing age, neutrophil-lymphocyte ratio (NLR), and serum creatinine levels have been reported to have a significant correlation with in-hospital and short-term mortality. In STEMI, an excessive inflammatory response occurs as a result of early ischaemia. Therefore, inflammatory markers have recently become the focus of attention in the assessment of severity in the early stages of STEMI^[5,6]^.

The immature granulocytes (IG) count, which is a practical marker of local and systemic inflammation, can be quickly and easily obtained using a complete blood count (CBC) by means of recent technological advances such as automated blood cell analysers. The IG count can reflect the fraction of circulating IG without demanding extra costs or time^[7-9]^. A literature review has shown that only a few studies have investigated the association between the IG count and the severity of STEMI. Therefore, this study aimed to evaluate the significance of the IG count as a prognostic marker in STEMI.

## METHODS

Ethics committee approval was obtained with decision date and number was 1/10/2020 and 15/6 before starting the study. Data from patients who were admitted to the emergency department with chest pain, admitted in the coronary care unit with a STEMI diagnosis, and who underwent primary percutaneous coronary intervention (pPCI) within the period from January 1^st^ 2019 to January 1^st^ 2020, were reviewed retrospectively. The respective data were retrieved and documented from the hospital automation system file. Criteria provided by international cardiology societies were used to make the diagnosis of STEMI^[10]^. Patients’ age, gender, haemogram, biochemistry results and mortality were recorded. A total of 146 patients aged ≥18 years meeting the study inclusion criteria were enrolled in the study. Patients under 18 years old, pregnant women, patients with myeloproliferative and chronic inflammatory diseases, kidney disease, liver disease, and malignancies, patients with missing information in medical records, patients referred to an external centre, and patients who refused treatment were excluded from the study. Twenty-one patients were excluded from the study as per the above criteria.

All patients included in the study underwent pPCI as an indicator of revascularization. No patient included in the study was referred to the vascular surgery department to undergo emergency coronary artery bypass grafting (CABG) surgery. Before the intervention, patients were given 300 mg of aspirin and 300 mg of clopidogrel or 180 mg of ticagrelor and low-molecular-weight heparin as per guidelines. CBCs were performed in all patients within two hours of their emergency admission. From the haemogram parameters, white blood cells (WBC), haemoglobin (Hb), platelets (Plt), mean platelet volume (MPV), neutrophils, lymphocytes, and IG values were recorded. From the biochemical parameters, high-sensitive troponin-T (hsTnT), C-reactive protein (CRP), creatinine, and glucose levels measured in the emergency room and high-density lipoprotein (HDL) cholesterol, low-density lipoprotein (LDL) cholesterol, total cholesterol, and triglyceride levels measured within 24 hours after hospital admission were recorded. The IG count, as a routine parameter in CBC, was measured using a Sysmex XN-1000 modular system (Sysmex, Kobe, Japan). In-hospital mortality rates were examined. Left ventricular ejection fraction (LVEF) was recorded based on echocardiography reports. Killip classification system classes recorded at the time of admission were recorded and accepted as baseline characteristics. Patients were divided into two groups as patients with low (<0.6) and high (≥0.6) IG counts and compared.

### Statistical Analysis

Statistical analyses were performed using SPSS version 21.0 (SPSS Inc., Chicago, IL, USA). Continuous variables were expressed as mean±standard deviation and categorical variables were expressed as numbers (n) and percentages (%). Categorical data were analysed using the chi-square test. Regarding the conformity of data to a normal distribution, the independent t-test or the Mann-Whitney U test was used, when appropriate. The optimum cut-off value of the IG count in predicting in-hospital mortality in patients with STEMI was evaluated by the receiver operating characteristic (ROC) analysis. Variables that could act on mortality were evaluated by logistic regression analysis. A *P*-value of <0.05 was considered statistically significant.

## RESULTS

A total of 146 patients who met the inclusion criteria were included in the study. Of these patients, 112 (76.7%) were men and 34 (33.3) were women. The mean age of the patients was 62.65±14.06 years. Mean ejection fraction (EF) was 44% (30-52%), mean systolic blood pressure (SBP) was 140 (80-160), and mean diastolic blood pressure (DBP) was 85 (60-95). Seventy-four (50.7) patients met the class I criteria according to the Killip classification. The most common myocardial infarction (MI) types were inferior (46.6%) and anterior (44.5%) MI in decreasing order of frequency. Hypertension, dyslipidaemia and diabetes were the most common risk factors. The mean WBC count was 11.98±4.89. The mean CRP and IG values were 35.16±18.75 and 0.61±0.51, respectively. Table 1 presents the baseline clinical characteristics of patients with STEMI. When patients were divided into groups according to in-hospital mortality, 123 (84.2%) were in the survivor group and 23 (15.8%) were in the non-survivor group. The mean age of patients was found to be significantly higher in the non-survivor group (74.86±13.14 *vs.* 60.36±13.06, *P*<0.001). The number of male patients was significantly higher in the survivor group (*P*=0.002). Mean SBP, DBP, heart rate (HR) values and the percentage of patients in Killip class I were significantly higher in the survivor group compared to the non-survivor group (*P*<0.05 for all markers). Mean WBC, neutrophils, glucose, blood urea nitrogen (BUN), creatinine, alanine transaminase (ALT) and troponin levels were significantly higher but the mean haemoglobin levels were significantly lower in non-survivors compared to survivors (*P*<0.05 for all markers). Mean IG counts were significantly higher in non-survivors compared to survivors (1.12±0.22 *vs.* 0.50±0.28, *P*<0.001). The comparison of demographic data and laboratory values between groups are presented in Table 1.

**Table 1:** Clinical and demographic characteristics of the study population.

Variables	Survivors (n=123)	Non-survivors (n=23)	*P*-value
Age (years)	60.36±13.06	74.86±13.14	<0.001
Male gender, n(%)	100 (81.3)	12 (52.2)	0.002
SBP, mmHg	146 (85-172)	110 (70-140)	<0.001
DBP, mmHg	90 (70-110)	72 (62-82)	0.003
Heart rate, beats/min	82.58±18.73	85.47±25.28	0.411
Ejection fraction, %	45 (40-60)	35 (30-54)	<0.001
Killip class			<0.001
I	73 (59.3)	1 (4.3)	
II	34 (27.6)	2 (8.7)	
III	15 (12.2)	12 (52.2)	
IV	1 (0.9)	8 (34.8)	
Type of MI			0.389
Anterior MI	55 (44.7)	10 (43.5)	
Inferior MI	55 (44.7)	13(56.5)	
Posterior and RV MI	9 (7.3)	0 (0)	
High lateral MI	4 (3.3)	0 (0)	
Previous history			
Hypertension	64 (52.0)	15 (65.2)	0.244
Diabetes mellitus	39 (31.7)	10 (43.5)	0.273
Dyslipidemia	44 (35.8)	11 (47.8)	0.274
History of CAD	38 (30.9)	6 (26.1)	0.645
Laboratory findings			
WBC count (×10^3^/mm^3^)	11.42±4.15	14.96±7.15	0.021
Neutrophils (×10^3^/mm^3^)	7.73±3.61	11.33±6.91	<0.001
Lymphocytes (×10^3^/mm^3^)	2.61±1.74	2.65±2.11	0.777
Hemoglobin (mg/dL)	14 (12-15)	12 (9-14)	<0.001
Glucose (mg/dL)	162.08±92.86	232.47±28.05	<0.001
BUN	19.10±10.17	30.91±4.22	0.002
Creatinine (mg/dL)	1.1 (0.5-1.2)	1.2 (0.7-1.3)	<0.001
Alanine transaminase (IU/L)	28.31±19.86	93.43±37.58	0.027
IG%	0.50±0.28	1.12±0.22	<0.001
CRP (mg/dL)	21.22±7.48	37.62±22.04	0.327
Troponin T (ng/L)	644.71±148.75	1610±448.15	0.003
Lipid profiles (mg/dL)			
Triglycerides	120.61±95.58	160.21±29.56	0.131
Total cholesterol	204.95±53.33	193.86±63.83	0.273
High-density lipoprotein	44.43±10.98	42.65±12.49	0.509
Low-density lipoprotein	140.63±57.22	117.17±42.69	0.118

BUN=blood urea nitrogen; CRP=C-reactive protein; DBP=diastolic blood pressure; IG=immature granulocytes; MI=myocardial infarction; RV=right ventricular; SBP=systolic blood pressure; WBC=white blood cells

When patients were divided into two groups as those with low (<0.6) and high (≥0.6) IG counts, it was observed that there were 94 (64.3%) and 52 (35.7%) patients in the low and high IG groups, respectively. Patients with high IG counts had significantly lower SBP and DBP values. Laboratory analysis results showed that patients in the high IG count group had significantly higher WBC (14.41±6.38 *vs.* 10.63±3.14, *P*<0.001), neutrophil (10.23±5.99 *vs.* 7.23±2.84, *P*<0.001), glucose (193.41±94.95 *vs.* 161.97±94.99, *P*=0.009), BUN (23.50±13.79 *vs.* 19.56±12.55, *P*=0.03) and creatinine (1.34±0.71 *vs.* 1.11±0.54, *P*=0.001) levels compared to the low IG count group. Furthermore, mortality rates were significantly higher in the high IG count group compared to the low IG count group (26.9% *vs.* 9.6%, *P*=0.006) (Table 2).

**Table 2:** Clinical and demographic characteristics of the study population.

Variables	Low-IG group (<0.6, n=94)	High-IG group (≥0.6, n=52)	*P*-value
Age (years)	62.54±13.63	63.00±14.96	0.997
Male gender, n(%)	73 (77.7)	39 (75)	0.564
SBP, mmHg	147 (130-155)	128 (120-136)	0.001
DBP, mmHg	90 (80-112)	79 (69-88)	<0.001
Heart rate, beats/min	83 (80-114)	82 (72-96)	0.811
Ejection fraction, %	44 (40-60)	41 (40-55)	0.181
Killip class			0.052
I	50 (53.2)	24 (46.2)	
II	27 (28.7)	9 (17.3)	
III	14 (14.9)	13 (25)	
IV	3 (3.2)	6 (11.5)	
Type of MI			0.071
Anterior MI	46 (48.9)	19 (36.5)	
Inferior MI	37 (39.4)	31 (59.6)	
Posterior and RV MI	7 (7.4)	2 (3.9)	
High lateral MI	4 (4.3)	0 (0)	
Previous history			
Hypertension	52 (55.3)	27 (51.9)	0.693
Diabetes mellitus	31 (33.0)	18 (34.6)	0.491
Dyslipidemia	34 (36.2)	21 (40.4)	0.615
History of CAD	26 (27.7)	18 (34.6)	0.380
Laboratory findings			
WBC count (×10^3^/mm^3^)	10.63±3.14	14.41±6.38	<0.001
Neutrophils (×10^3^/mm^3^)	7.23±2.84	10.23±5.99	<0.001
Lymphocytes (×10^3^/mm^3^)	2.43±1.49	2.97±2.23	0.223
Hemoglobin (mg/dL)	13 (12-15)	13 (12.5-15)	0.134
Glucose (mg/dL)	161.97±94.99	193.41±94.95	0.009
BUN	19.56±12.55	23.50±13.79	0.030
Creatinine (mg/dL)	1.11±0.54	1.34±0.71	0.001
Alanine transaminase (IU/L)	29.31±20.38	55.28±17.19	0.365
CRP (mg/dL)	43.67±28.61	19.05±3.87	0.324
Troponin T (ng/L)	616.93±120.61	1117.15±352.10	0.739
Lipid profiles (mg/dL)			
Triglycerides	116.46±73.31	145.61±19.98	0.400
Total cholesterol	201.53±52.32	206.25±60.01	0.859
High-density lipoprotein	43.41±9.69	45.51±13.51	0.545
Low-density lipoprotein	133.11±45.82	143.84±70.16	0.710
Mortality	9 (9.6)	14 (26.9)	0.006

BUN=blood urea nitrogen; CRP=C-reactive protein; DBP=diastolic blood pressure; IG=immature granulocytes; MI=myocardial infarction; RV=right ventricular; SBP=systolic blood pressure; WBC=white blood cells

**Fig. 1:**
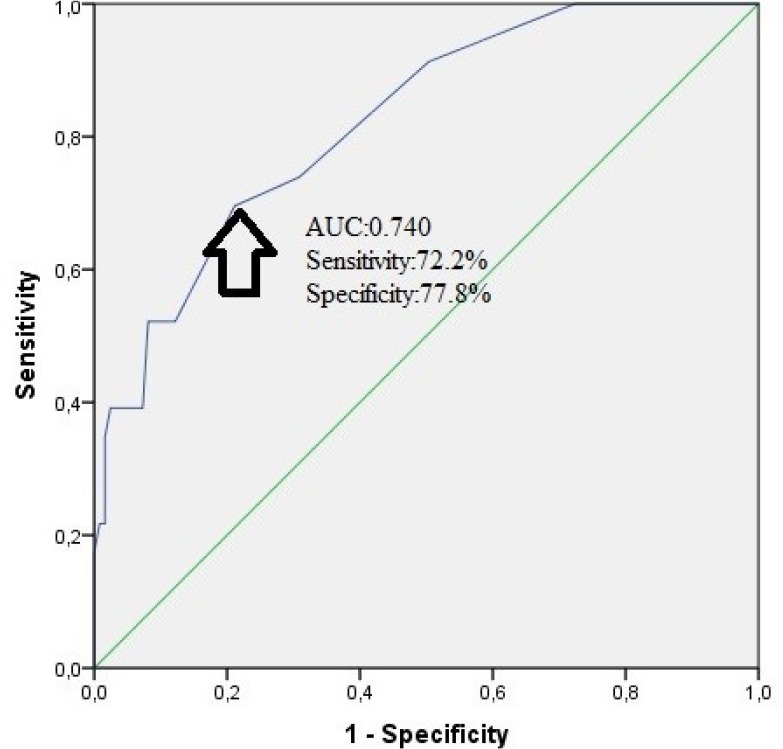
*Receiver operating characteristic (ROC) curve for immature granulocytes for predicting mortality in ST-segment elevation myocardial* infarction.

The multivariate logistic regression analysis revealed that age (OR: 7.486, 95% CI: 1.995-28.081, *P*=0.003), anaemia (OR: 1.634, 95% CI: 1.167-2.405, *P*=0.016), LVEF (OR: 0.385, 95% CI: 0.270-0.825, *P*=0.017), Killip class (OR: 6.382, 95% CI: 2.091-14.505, *P*=0.017), and IG count (OR: 5.003, 95% CI: 1.426-7.557, *P*<0.001) were the independent predictors of in-hospital mortality (Table 3). Furthermore, in ROC curve analysis, the IG count was shown to predict in-hospital mortality with a sensitivity of 72.2% and specificity of 77.8% at a cut-off value of 0.65 (area under the curve: 0.740, 95% CI: 0.635-0.846, *P*<0.001, Figure 1).

**Table 3:** Logistic regression analysis of the independent predictors of mortality.

	Univariate analysis		Multivariate analysis	
Variables	OR (95% CI)	P	OR (95% CI)	*P*-value
Age	7.200 (2.496-20.789)	<0.001	7.486 (1.995-28.081)	0.003
Male sex	0.251 (0.098-0.639)	0.004	0.455 (0.129-1.610)	0.222
LVEF (%)	0.321 (0.232-0.550)	0.003	0.385 (0.270-0.825)	0.017
Anemia	1.233 (1.128-2.124)	0.033	1.634 (1.167-2.405)	0.016
Killip class >1	8.211 (4.194-16.050)	0.001	6.382 (2.091-14.505)	0.017
IG (%)	3.480 (1.386-8.736)	<0.001	5.003 (1.426-7.557)	0.012

CRP=C-reactive protein; IG=immature granulocytes; LVEF=left ventricular ejection fraction

## DISCUSSION

Early assessment and treatment are of great importance in patients with cardiovascular disease associated with high mortality, such as STEMI^[11]^. In previous studies, several biomarkers, including troponin, CRP, N-terminal pro-brain natriuretic peptide (NT-proBNP) and NLR, as well as clinical scoring, were used as prognostic indicators. A haemogram, which is a simple test that can be easily evaluated by all physicians, is requested in almost all patients who are admitted to an emergency department or intensive care unit. The IG count is less known among physicians, but it is a simple haemogram parameter. Several studies have recently suggested that the IG count can be used to predict both the short- and long-term mortality associated with many diseases^[12,13]^. The present study has shown that the IG count is an independent risk factor that can be used to predict the prognosis in patients with STEMI.

The association between MI and inflammation has been known for many years. Furthermore, inflammation is closely associated with the prognosis and possible complications in patients with STEMI^[14]^. Increasing intensity of inflammation increases the likelihood that atherosclerotic plaques can lead to MI^[15]^. In many previous studies, haemogram parameters (*e.g.* WBC, neutrophil counts, etc.) and the ratios of such parameters to each other have been reported to have a prognostic value in patients with coronary artery disease and STEMI^[16-19]^. The present study has shown an association between the IG count, a simple haemogram parameter, and poor prognosis in STEMI patients.

The IG count shows the number of serial myelocytic cells in the peripheral blood and can be obtained by means of automated blood cell analysers^[20]^. Inflammation and trauma are known to lead to the occurrence of circulating immature cells that normally should not be present in the peripheral blood. Therefore, the diagnostic and prognostic values of circulating immature cells in sepsis, trauma, and gastrointestinal system diseases have been discussed in many studies^[12,20,21]^. Krishnan et al.^[22]^ reported that the IG count was an effective marker in predicting the severity of the infection and determining the need for early intervention in critically ill patients. In another recent study that we have performed recently, we have reported that the IG count was associated with mortality in upper gastrointestinal system diseases and that the IG count helped predict mortality with 66.7% sensitivity and 75.7% specificity at a cut-off value of 0.95^[7]^.

Park et al.^[23]^ studied the diagnostic value of the IG count and its role in estimating the occurrence of complications in patients with acute appendicitis. In that study, it was shown that the IG count was not as effective as other inflammatory markers in making the diagnosis and estimating the occurrence of complications. Sinaga et al.^[13]^ have reported that the IG count obtained at the time of admission to the emergency department serves as a marker, which could effectively help predict the 30-day mortality with a cut-off value of 1.05 in patients with peritonitis. Huang et al.^[24]^ reported that the IG count could be a predictor of possible lung complications at the onset of acute pancreatitis. In another recent study, haematological biomarker levels measured at the time of admission of patients with acute MI were investigated as predictors of all-cause mortality. This study found that an IG count >0.3 was significantly correlated with mortality^[25]^. Similarly, in the present study, we have observed a significant association between high IG values and mortality in patients with STEMI. Our study results show that the IG count helps to predict in hospital mortality with 72.2% sensitivity and 77.8% specificity at a cut-off value of 0.65.

### Limitations

This study has several limitations. First, it was designed as a single-centre, retrospective study. Second, the time elapsed from the onset of symptoms to the time of sampling and the time from diagnosis of STEMI to pPCI were not analysed, which might have affected our results. Lastly, serial IG counts could not be performed, and IG counts could not be compared with the levels of some inflammatory parameters such as tumour necrosis factor and interleukin 6. There is a need for prospective multi-centre studies to show that the IG count can be used as a prognostic marker in patients with STEMI.

## CONCLUSION

A high IG count in the blood collected at the time of admission to the emergency room is a predictor of mortality in patients with STEMI.
